# Interhemispheric Inhibition Induced by Transcranial Magnetic Stimulation Over Primary Sensory Cortex

**DOI:** 10.3389/fnhum.2016.00438

**Published:** 2016-08-31

**Authors:** Yasuyuki Iwata, Yasutomo Jono, Hiroki Mizusawa, Atsushi Kinoshita, Koichi Hiraoka

**Affiliations:** ^1^Graduate School of Comprehensive Rehabilitation, Osaka Prefecture UniversityHabikino, Japan; ^2^College of Health and Human Sciences, Osaka Prefecture UniversityHabikino, Japan

**Keywords:** interhemispheric inhibition, primary sensory area, primary motor area, somatosensory input, transcranial magnetic stimulation

## Abstract

The present study investigated whether the long-interval interhemispheric inhibition (LIHI) is induced by the transcranial magnetic stimulation over the primary sensory area (S1-TMS) without activation of the conditioning side of the primary motor area (M1) contributing to the contralateral motor evoked potential (MEP), whether the S1-TMS-induced LIHI is dependent on the status of the S1 modulated by the tactile input, and whether the pathways mediating the LIHI are different from those mediating the M1-TMS-induced LIHI. In order to give the TMS over the S1 without eliciting the MEP, the intensity of the S1-TMS was adjusted to be the sub-motor-threshold level and the trials with the MEP response elicited by the S1-TMS were discarded online. The LIHI was induced by the S1-TMS given 40 ms before the test TMS in the participants with the attenuation of the tactile perception of the digit stimulation (TPDS) induced by the S1-TMS, indicating that the LIHI is induced by the S1-TMS without activation of the conditioning side of the M1 contributing to the contralateral MEP in the participants in which the pathways mediating the TPDS is sensitive to the S1-TMS. The S1-TMS-induced LIHI was positively correlated with the attenuation of the TPDS induced by the S1-TMS, indicating that the S1-TMS-induced LIHI is dependent on the effect of the S1-TMS on the pathways mediating the TPDS at the S1. In another experiment, the effect of the digit stimulation given before the conditioning TMS on the S1- or M1-TMS-induced LIHI was examined. The digit stimulation produces tactile input to the S1 causing change in the status of the S1. The S1-TMS-induced LIHI was enhanced when the S1-TMS was given in the period in which the tactile afferent volley produced by the digit stimulation just arrived at the S1, while the LIHI induced by above-motor-threshold TMS over the contralateral M1 was not enhanced by the tactile input. Thus, the S1-TMS-induced LIHI is dependent on the status of the S1 modulated by the tactile input, and the pathways mediating the sub-motor-threshold S1-TMS-induced LIHI are not the same as the pathways mediating the above-motor-threshold M1-TMS-induced LIHI.

## Introduction

Interhemispheric inhibition (IHI) is the neural mechanism that causes the inhibition of one hemisphere in response to the activation of another. The IHI between the primary motor cortices (M1s) has been observed through giving the transcranial magnetic stimulation (TMS) over the primary motor area (M1); the conditioning TMS over the M1 inhibits the test motor evoked potential (MEP) in the hand muscle ipsilateral to the conditioning TMS side (Ferbert et al., 1992; Meyer et al., 1995). Based on these previous findings, it is rational to suppose that the IHI between the M1 and contralateral primary sensory area (S1) may also be tested by observing the inhibition of the test MEP induced by the TMS over the S1 contralateral to the test TMS side.

A previous study reported a negative finding on this issue; the sub-motor-threshold transcranial magnetic stimulation over the primary sensory area (S1-TMS) given 50 ms before test TMS over the contralateral M1 did not modulate the test MEP in the first dorsal interosseous (FDI) muscle (Mochizuki et al., 2004). In contrast, a recent study reported a positive finding on that; the above-motor-threshold S1-TMS given from 30 to 50 ms before the test TMS over the contralateral M1 inhibited the test MEP, indicating that the above-motor-threshold S1-TMS induces the long-interval interhemispheric inhibition (LIHI; Ni et al., 2009).

The reason for the conflicting findings between the study by Mochizuki et al. (2004) and Ni et al. (2009) must be the intensity of the S1-TMS. In some experiments in the previous study by Ni et al. (2009), the LIHI was induced by the S1-TMS eliciting the MEP with 1 mV of amplitude. Simultaneously, in another experiment in their study, the S1-TMS-induced LIHI had the positive correlation with the amplitude of the MEP elicited by the S1-TMS, and the IHI was almost absent when the amplitude of the MEP elicited by the S1-TMS was zero in accordance with the regression line of the IHI as a function of the amplitude of the MEP elicited by the S1-TMS. Based on these findings, it seems to be likely that the S1-TMS-induced LIHI is dependent on the amplitude of the MEP elicited by the S1-TMS and is almost absent when the S1-TMS is given at the intensity below the motor threshold. Accordingly, it is possible to speculate that the LIHI induced by the S1-TMS is present only when the S1-TMS activates the ipsilateral M1 contributing to the MEP through the current spread to the M1 produced by the S1-TMS. In other words, the S1-TMS-induced LIHI may be due to activation of some interneurons in the M1 induced by the current spread caused by the S1-TMS.

In the present study, an investigation was made to elucidate whether the LIHI is induced by the S1-TMS without activation of the conditioning side of the M1 contributing to the contralateral MEP. We expect that the LIHI induced by the S1-TMS without activation of the conditioning side of the M1 contributing to the contralateral MEP is not apparent when it is estimated across healthy adult humans, based on the negative findings on the S1-TMS-induced LIHI in the study by Mochizuki et al. (2004) and Ni et al. (2009). In spite of these negative previous findings, the LIHI induced by the S1-TMS without activation of the conditioning side of the M1 contributing to the contralateral MEP may be apparent when estimating the LIHI in the participants who are sensitive to the S1-TMS. The magnitude of the LIHI was greater as the amplitude of the MEP elicited by the S1-TMS was larger in the previous study by Ni et al. (2009). This may reflect a fact that the LIHI is only present when the intensity of the S1-TMS is strong enough to change the status of the S1. Thus, the previous negative findings may be explained by a view that the LIHI is present only when the S1-TMS effectively changes the status of the S1. The effect of the S1-TMS on the tactile perception of the digit stimulation (TPDS) must reflect the effect of the S1-TMS on the interneurons in the S1, because some pathways mediating the TPDS originate from the S1 (Porro et al., 2004; Dijkerman and de Haan, 2007). Thus, in spite of the negative findings in the previous studies, the S1-TMS-induced LIHI may be present when estimating that in healthy adult humans in whom the TPDS is sensitive to the S1-TMS. Based on this view, the S1-TMS-induced LIHI was estimated both in all of the participants and in the participants in whom the TPDS was sensitive to the S1-TMS in the present study.

In the present study, S1-TMS-elicited MEP was strictly excluded, in order to investigate whether the LIHI is induced by the S1-TMS without activation of the conditioning side of the M1 contributing to the contralateral MEP. That is, the S1-TMS was given at the intensity at which the contralateral MEP response larger than 50 μV was not elicited in 10 consecutive preliminary trials, and the trials with the MEP response larger than 50 μV were discarded online.

Another question is whether the S1-TMS-induced LIHI is dependent on the status of the S1 modulated by the tactile input. The change in the activity of the left S1 induced by the TMS over the right parietal cortex (2–4 cm posterior to the hotspot of the muscle representation) was modulated by the electrical stimulus over the right median nerve (MN) projecting to the left S1 (Blankenburg et al., 2008). The site 2–4 cm posterior to the hotspot of the muscle representation has been considered to be the appropriate TMS site of the S1 (Harris et al., 2002; McKay et al., 2003; Koch et al., 2006; Palomar et al., 2011). Accordingly, the finding by Blankenburg et al. (2008) means that the interhemispheric interaction between the S1s is dependent on the state of the S1 that receives the interhemispheric input. However, the effect of the tactile input to the conditioning side of the S1 on the S1-TMS-induced LIHI has not been investigated.

In order to investigate the effect of the tactile input to the conditioning side of the S1 on the S1-TMS-induced LIHI, the status of the conditioning side of the S1 was modulated by the tactile stimulation to the digit (DS). Generally, the MN is stimulated at the wrist in order to induce the sensory evoked potential (SEP) in the contralateral hemisphere (Nuwer et al., 1994). When the MN is electrically stimulated at the wrist, both the cutaneous and muscle afferents are stimulated, but the cutaneous afferents are mainly stimulated when the digit is stimulated (Chen et al., 1999). Muscle afferent projects to the areas 3a and 2, but cutaneous afferents project to the area 3b and 1 (Friedman and Jones, 1981). Thus, in the present study, electrical stimulation was given over the digit so that the status of the limited areas of the S1 was modulated by the tactile input.

In addition, the effect of the DS on the LIHI induced by the above-motor-threshold M1-TMS was also examined as the control experiment for the effect of the DS on the S1-TMS-induced LIHI. If the S1-TMS-induced LIHI is modulated by the DS, but the above-motor-threshold M1-TMS-induced LIHI is not, the S1-TMS-induced LIHI and M1-TMS-induced LIHI must be mediated by different mechanisms. Given this is true, the finding indirectly supports our view that the LIHI is induced by the S1-TMS without activating the conditioning side of the M1 contributing to the MEP.

The other question is whether the pathways mediating the TPDS at the S1 interact with the pathways mediating the LIHI. The S1-TMS induces attenuation of the TPDS (Cohen et al., 1991; Seyal et al., 1992, 1995), indicating that the S1-TMS interferes the TPDS. Thus, the S1-TMS-induced LIHI must be dependent on the effect of the S1-TMS on the pathways mediating the TPDS at the S1, if the pathways mediating the S1-TMS-induced LIHI interact with the pathways mediating the TPDS at the S1. We tested this hypothesis as well.

## Experiment 1

### Materials and Methods

#### Participants

Seventeen healthy humans aged 28.8 ± 1.4 years (10 males and 7 females) participated in Experiment 1 (Table 1). The participants had no history of neurological disease. All participants were right-handed according to the Edinburgh Handedness Inventory (Oldfield, 1971). All participants gave written informed consent for study participation prior to the experiment. The experiment was approved by the ethics committee of Osaka Prefecture University.

**Table 1:** **Applied exclusion criteria**.

	Test MEP size (0.5–1.5 mV)	TP ratio (<1.0)	*N*
Experiment 1			17
TP ratio			17
S1-TMS-induced IHI	✓		14
Correlation between IHI and TP ratio	✓		14
S1-TMS-induced IHI (sub-group analysis)	✓	✓	10
DS effect on test MEP	✓	✓	10
DS effect on S1-TMS-induced IHI	✓	✓	10
Experiment 2			13
TP ratio			13
DS effect on S1-TMS-induced LIHI	✓		13
DS effect on S1-TMS-induced			
LIHI (sub-group analysis)	✓	✓	10
DS effect on M1-TMS-induced			
LIHI (sub-group analysis)	✓	✓	9

#### DS

**Figure 1:**
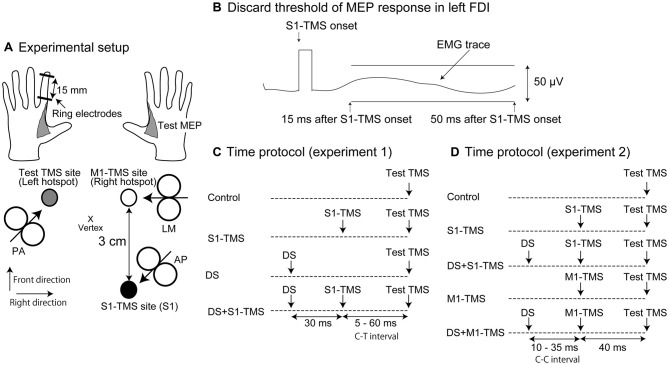
**Experimental setup (A), the discard threshold of the motor evoked potential (MEP) response in the left first dorsal interosseous (FDI) muscle (B) and time protocol of the test and conditioning stimuli in Experiments 1 (C) and 2 (D).** The arrow in each coil indicates the direction of the electrical current in the brain **(A)**. The deviation of the electromyographic (EMG) trace in the time window between 15 and 50 ms after the transcranial magnetic stimulation over the primary sensory area (S1-TMS) larger than 50 μV is considered to be the presence of the MEP response and the trials with the EMG trace larger than this size of the deviation are discarded from data analysis **(B)**. MEP, motor evoked potential; FDI, first dorsal interosseous; S1, primary sensory area; TMS, transcranial magnetic stimulation; AP, anterior-posterior position; PA, posterior-anterior position; LM, lateral-medial position; DS, tactile stimulation to the index finger; M1, primary motor area; C-T interval, conditioning-testing interval; C-C interval, conditioning-conditioning interval.

Electrical stimulation was given over the left index finger for inducing the tactile input to the S1 producing the tactile perception. A pair of ring electrodes that electrically elicits a tactile sensation was braced over the left index finger as shown in Figure 1A (SL-100-1; Unique Medical, Tokyo, Japan). The anode of the electrodes was braced over the distal phalanx, and the cathode of the electrodes was braced over the intermediate phalanx. The distance between the electrodes was 15 mm. A monophasic square-wave pulse of 1 ms was generated by an electrical stimulator (SS-104J; Nihon Kohden, Tokyo, Japan). The perceptual threshold was defined as the minimum intensity at which the participant perceived tactile sensation nine times or more out of 10 consecutive DSs.

#### Electromyographic Recordings

The Ag/AgCl surface electrodes recording electromyographic (EMG) activity were placed over the left and right FDI muscles configured in belly-tendon montages. The EMG signals were amplified by an EMG amplifier (MEG-2100; Nihon Kohden, Tokyo, Japan) with a band-pass filter from 15 Hz to 3 kHz. The amplified EMG signals were converted to digital signals at a sampling rate of 10 kHz using an A/D converter (PowerLab 800 s; ADInstruments, Colorado Springs, CO, USA), and the digital signals were stored on a personal computer.

#### Test TMS

The test TMS was given over the hotspot of the right FDI muscle representation using a figure-of-eight-shaped coil (YM-131B; Nihon Kohden) connected to a magnetic stimulator (SMN-1200; Nihon Kohden). The coil had an outer diameter of 99 mm for one half of the coil. The maximum intensity of the coil was 1.03 T. The coil oriented to the direction in which the TMS induced an anterior-medial current in the brain (posterior-anterior position [PA]) as shown in Figure 1A (Ni et al., 2011). The coil was first placed over a site 3 cm left of the vertex and was moved little by little to find a hotspot where the largest test MEP response in the right FDI muscle was elicited. Then, the coil was positioned at the hotspot of the right FDI muscle representation, and the test TMS intensity that produced the test MEP response with a mean amplitude of around 1 mV was determined.

#### S1-TMS

The conditioning TMS over the right S1 (S1-TMS) was given using a figure-of-eight-shaped coil (YM-132B; Nihon Kohden) connected to another magnetic stimulator (SMN-1200; Nihon Kohden). The coil had an outer diameter of 110 mm for one half of the coil. The maximum intensity of the coil was 0.71 T. The coil oriented to the direction in which the TMS induced a posterior-lateral current in the brain (anterior-posterior position [AP]) as shown in Figure 1A (Ni et al., 2011). We used the AP position in order to maximize the S1-TMS intensity of the given intensity level in terms of the motor threshold (just below the motor threshold). The motor threshold of the MEP elicited by the coil in the AP position is higher than that elicited by the coil placed in the PA or lateral-medial position (LM; Volz et al., 2015). Thus, giving the TMS with the coil in the AP position more likely gives the higher intensity of the S1-TMS without eliciting the MEP response in the left FDI muscle comparing with giving the S1-TMS with the coil in the PA or LM position.

Previous studies have used different procedures to determine the site of the S1-TMS. In some studies, the C3 or C4 was targeted for the S1-TMS administration (Cohen et al., 1991; Seyal et al., 1992; Harris et al., 2002), while in others, a site 3 or 4 cm posterior to the hotspot of the muscle representation was targeted (McKay et al., 2003; Koch et al., 2006; Palomar et al., 2011). The site of the S1 has also been determined using anatomical MRI (Meehan et al., 2008; Ni et al., 2009). In the present study, the S1-TMS was administered to a site 3 cm posterior to the hotspot of the left FDI muscle representation. Therefore, before the site of the S1 could be determined, the hotspot of the left FDI muscle representation had to be located. For this purpose, we used the same procedure as used to search for the hotspot of the right FDI muscle representation. Finally, the intensity of the S1-TMS was determined as the maximum intensity at which the MEP response larger than 50 μV of the amplitude in the left FDI muscle was not observed across 10 consecutive S1-TMSs (Figure 1B). This amplitude was used as the cut-off level of the MEP response, because usually the presence and absence of the MEP response is discriminated at this level when estimating the motor threshold (Chen et al., 2008). If the MEP response larger than 50 μV was absent for the 10 consecutive trials, even at the maximum output, the S1-TMS was given with the intensity at the maximum output during the experimental session.

#### Time Course of IHI

The time course of the IHI induced by the S1-TMS, DS, or S1-TMS with the DS was examined. The intensity of the DS was 1.5 times the intensity at the perceptual threshold. The S1-TMS was given 5, 10, 20, 40, or 60 ms before the test TMS under the S1-TMS condition (Figure 1C). The interval between the test TMS and S1-TMS (conditioning-testing [C-T] interval) was altered randomly on a trial-by-trial basis. The S1-TMS was given 5, 10, 20, 40, or 60 ms before the test TMS, and the DS was given 30 ms before the S1-TMS under the DS + S1-TMS condition. Thus, the DS was given 35, 40, 50, 70, or 90 ms before the test TMS under the DS condition. The number of trials was 10 under each condition with each C-T interval. Only the test TMS was given under the control condition. The trials under the control condition were inserted between the trials under the DS, S1-TMS, and DS + S1-TMS conditions. The number of trials inserted under the control condition was 10. The total number of planned trials in this experimental session was 160: 50 trials each under the S1-TMS, DS + S1-TMS, and DS conditions; and 10 trials under the control condition. The participants closed their eyes with their hands relaxed throughout the experiment. A trigger of the TMS was given when background EMG activity was absent. The inter-trial interval was 10–20 s. The pre-stimulus background EMG burst was identified visually, and the trials accompanied by this burst were discarded online. In addition, the trials in which the trials with the MEP response larger than 50 μV of the amplitude in the left FDI muscle were discarded online. Then, complementary trials for the discarded trials were conducted after the completion of the planned trials.

#### Attenuation of TPDS by S1-TMS

Attenuation of the TPDS induced by the S1-TMS was tested in previous studies (Cohen et al., 1991; Seyal et al., 1992, 1995). This reflects the effect of the S1-TMS on the pathways mediating the TPDS at the S1. The TPDS when both the DS and S1-TMS were given (DS + S1-TMS condition) was compared with that when only the DS was given (DS condition) in this experimental session. The DS was given with an intensity at the perceptual threshold. Under the DS + S1-TMS condition, both the S1-TMS and DS were given (Figure 1C). The DS was given 30 ms after the S1-TMS under the DS + S1-TMS condition, because the prominent attenuation of the TPDS induced by the S1-TMS was present in this interval in our preliminary trials, and the maximum attenuation of the TPDS induced by the S1-TMS appeared when the DS was given 20–30 ms before the S1-TMS in a previous study (Seyal et al., 1992). Under the S1-TMS condition, only the S1-TMS was given. Under the DS condition, only the DS was given. The experimental condition was randomly altered on a trial-by-trial basis. Ten trials were conducted for each condition. The total number of trials was 30. The participants closed their eyes with their hands relaxed throughout the experiment. The participants were instructed to answer “yes” if they perceived a tactile stimulus and “no” or “weak” if they did not perceive it or if the sensation weakened after each DS in each trial. The TP ratio representing the attenuation of the tactile input induced by the S1-TMS was estimated; the probability of the TPDS under the S1-TMS + DS condition was divided by the probability of the TPDS under the DS condition. The TP ratio less than 1.0 indicates that the TPDS is attenuated by the S1-TMS.

#### Data Analysis

The MEP amplitude was estimated on a peak-to-peak basis. The TMS intensity was preliminarily determined so that the test MEP amplitude in the control condition was around 1 mV. This procedure was conducted to rule out the across-participant variability in the sensitivity of the test MEP to the conditioning stimulus. Nevertheless, the test MEP amplitude under the control condition in the experimental session may deviate from the target amplitude. Thus, participants in whom the mean amplitude of the test MEP under the control condition of the experimental session was outside the range of 0.5–1.5 mV were excluded from the analysis of the IHI. The TP ratio indirectly implies the effect of the TMS on the pathways mediating the TPDS at the S1. Thus, in the subgroup analysis, participants with the TP ratio equal to or more than 1.0 were excluded from the analysis of the IHI, in order to estimate the S1-TMS-induced IHI particularly in the participants in which the S1-TMS is effective on the pathways mediating the TPDS at the S1. The magnitude of the IHI was the test MEP amplitude under the S1-TMS condition expressed as a percentage of the test MEP amplitude under the control condition.

One-way ANOVA was conducted to test the difference in the test MEP amplitude among the control condition and five C-T intervals under the DS or S1-TMS condition, and to test the difference in the probability of the TPDS among the three experimental conditions. When one-way ANOVA revealed a significant difference among the means, it was followed by a multiple comparison test (Bonferroni test). Two-way ANOVA was conducted to test the difference in the change in the test MEP amplitude among the five C-T intervals and between the two experimental conditions. Pearson’s correlation coefficient between the magnitude of the S1-TMS-induced IHI and the TP ratio was estimated. A *t*-test was conducted for testing the difference between two means. The alpha level was 0.05. The results were expressed as means ± standard error.

### Results

#### Attenuation of TPDS by S1-TMS

**Figure 2:**
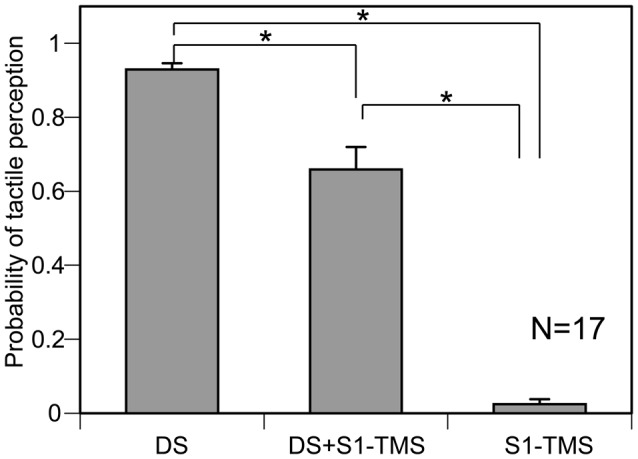
**The averaged probability of the tactile perception of the digit stimulation (TPDS) across 17 participants.** Bars indicate means, and error bars indicate standard errors of means. Asterisks indicate significant differences (*p* < 0.05). S1, primary sensory area; TMS, transcranial magnetic stimulation; DS, tactile stimulation to the index finger.

The probability of the TPDS under each condition across 17 participants is shown in Figure 2. The S1-TMS attenuated the TPDS. The probability of the TPDS was 0.93 ± 0.02 under the DS condition, 0.66 ± 0.06 under the DS + S1-TMS condition, and 0.02 ± 0.01 under the S1-TMS condition. There was a significant difference in the probability among the three experimental conditions (*F*_(2,32)_ = 135.55, *p* < 0.01). A *post hoc* test revealed a significant difference in this probability between each pair of conditions (*p* < 0.05). The average of the TP ratio across the participants was 0.72 ± 0.07.

#### IHI Induced by S1-TMS

The hotspot of the right FDI muscle representation was 5.8 ± 0.2 cm left to and 1.0 ± 0.3 cm anterior to the vertex in 17 participants. The test TMS intensity was 80.2 ± 4.1% of the maximum stimulator output. The S1-TMS site was 5.6 ± 0.3 cm right to and 1.6 ± 0.2 cm posterior to the vertex. The intensity of the S1-TMS was 89.1 ± 2.3% of the maximum stimulator output. The intensity of the S1-TMS was not significantly correlated with the TP ratio (*r* = 0.09, *p* = 0.74).

**Figure 3:**
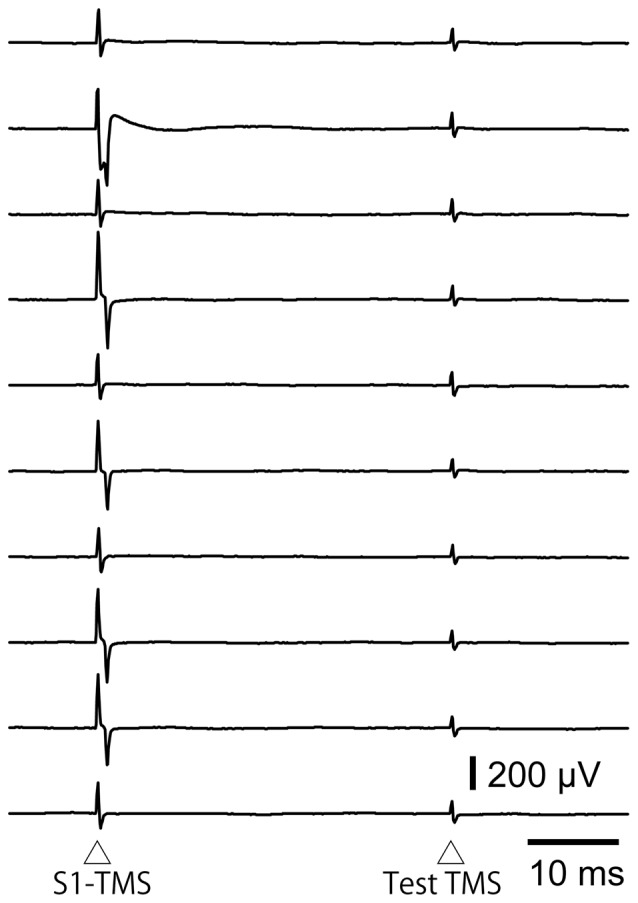
**The EMG traces in the left FDI muscle when the S1-TMS is given 40 ms after the test TMS in the trials included in the data analysis of the interhemispheric inhibition (IHI).** Note that no MEP response appears in these EMG traces. S1, primary sensory area; TMS, transcranial magnetic stimulation.

**Figure 4:**
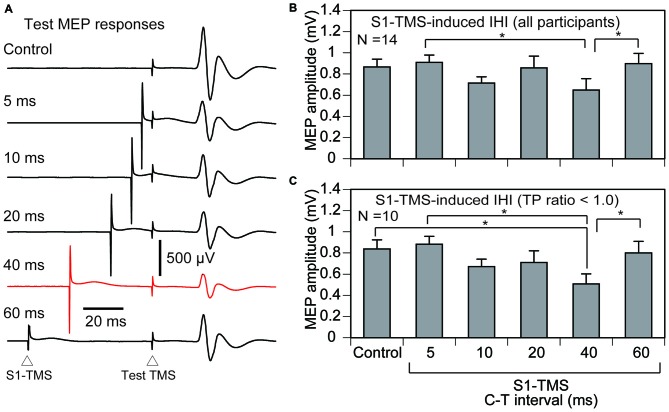
**The averaged test MEP responses (A), the averaged test MEP amplitudes under the S1-TMS and control conditions across the participants (B), and those in the participants with the TP ratio less than 1.0 (C).** The bar at the left side indicates the mean test MEP amplitude in the trials without the S1-TMS (control condition), and the other bars indicate the mean test MEP amplitudes in the trials with various C-T intervals of the S1-TMS (S1-TMS condition) **(B,C)**. Error bars indicate standard errors. Asterisks indicate significant differences (*p* < 0.05). MEP, motor evoked potential; S1, primary sensory area; TMS, transcranial magnetic stimulation; DS, tactile stimulation to the index finger; M1, primary motor area; C-T interval, conditioning-testing interval.

Three out of 17 the participants were excluded from the analysis of the IHI, because the average amplitude of the test MEP in the control trials in the experimental session was not within the range of 0.5–1.5 mV. Thus 14 participants (7 males and 7 females) were included in the analysis of the IHI (Table 1). The total number of trials in this experimental session was 182 ± 4. The average test MEP amplitude in the control trials in the experimental session was 0.87 ± 0.07 mV in these 14 participants. The MEP response in the left FDI muscle analyzed for the IHI was absent in all of the participants as shown in Figure 3. The average of the test MEP amplitudes with and without the S1-TMS in these 14 participants are shown in Figure 4B. The test MEP was small when the S1-TMS was given 40 ms before the test TMS (Figures 4A,B). One-way ANOVA revealed a significant difference in the test MEP amplitude among the control condition and the five C-T intervals under the S1-TMS condition (*F*_(5,65)_ = 3.90, *p* < 0.01). A *post hoc* analysis revealed that the test MEP amplitude under the S1-TMS condition with 40 ms of the C-T interval was significantly smaller than that in the S1-TMS condition with 5 and 60 ms of the C-T intervals (*p* < 0.05), but the test MEP amplitude under the S1-TMS condition with either of the C-T intervals was not significantly different from that under the control condition.

**Figure 5:**
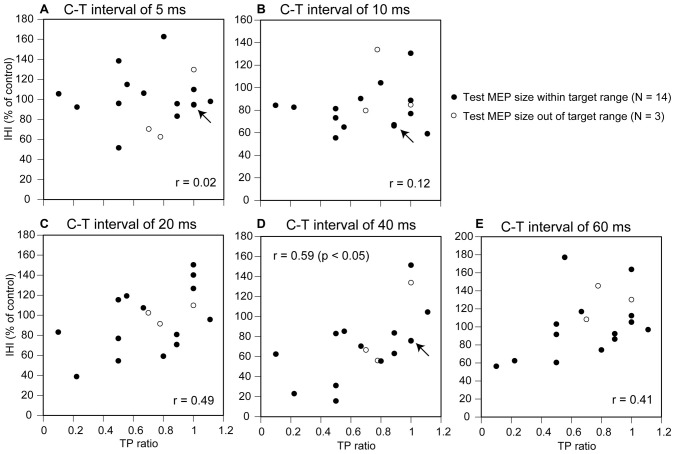
**Scatter plot of the IHI induced by the S1-TMS as a function of the TP ratio.** The panels indicate the plots for the C-T interval of 5 ms **(A)**, 10 ms **(B)**, 20 ms **(C)**, 40 ms **(D)**, and 60 ms **(E)**. Each arrow points two data points that superimpose at a same locus as if it is one. The magnitude of the IHI is the test MEP amplitude under the S1-TMS condition expressed as a percentage of the test MEP amplitude under the control condition. The data in the participants with the test MEP size out of target range (*N* = 3) are also plotted. Correlation coefficients are estimated for the participants with the test MEP size within the target range (*N* = 14). MEP, motor evoked potential; DS, tactile stimulation to the index finger; C-T interval, conditioning-testing interval.

The IHI induced by the S1-TMS as a function of the TP ratio in these 14 participants is shown in Figure 5. A moderate positive correlation between the IHI and TP ratio was observed when the S1-TMS was given with a C-T interval of 20, 40, or 60 ms. A significant positive correlation was observed between the TP ratio and IHI induced by the S1-TMS with a C-T interval of 40 ms (*p* < 0.05), but no such significant correlation was observed with the other C-T intervals.

Four out of 14 participants in whom the TP ratio was 1.0 or more were excluded from the sub-group analysis of the IHI of the participants with attenuation of the TPDS induced by the S1-TMS. That is, 10 participants were included in the sub-group analysis of the IHI (Table 1). The total number of trials in this experimental session was 179 ± 4. The average test MEP amplitude was 0.84 ± 0.09 mV in these 10 participants. The time course of the IHI induced by the S1-TMS in the participants with attenuation of the TPDS induced by the S1-TMS is shown in Figure 4C. The test MEP was small when the S1-TMS was given 40 ms before the test TMS (Figures 4A,C). One-way ANOVA revealed a significant difference in the test MEP amplitude among the control condition and the five C-T intervals of the S1-TMS condition (*F*_(5,45)_ = 5.66, *p* < 0.01). A *post hoc* test revealed that the test MEP amplitude under the S1-TMS condition with 40 ms of the C-T interval was significantly smaller than that in the S1-TMS condition with 5 or 60 ms of the C-T interval and was significantly smaller than that under the control condition (*p* < 0.05). The test MEP amplitude under the S1-TMS condition with a C-T interval of 40 ms was 39 ± 9% lower than that under the control condition.

#### Effect of DS on Test MEP

**Figure 6:**
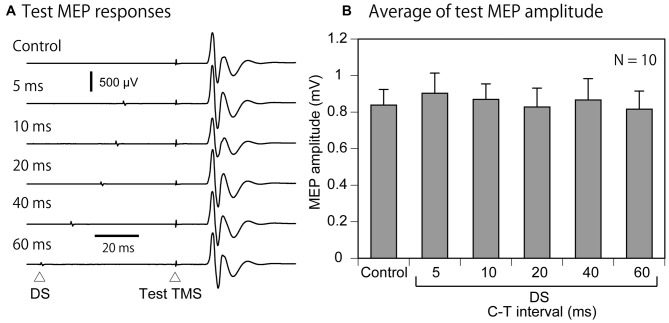
**The averaged MEP response (A) and the averaged effect of the DS on the test MEP in the participants with the TP ratio less than 1.0 (B).** The bar at the left side indicates the mean test MEP amplitude in the trials without the DS (control condition), and the other bars indicate the mean test MEP amplitudes in the trials with various C-T intervals of the DS (DS condition) **(B)**. Note that the C-T interval plus 30 ms is equal to the interval between the DS and test-TMS. Error bars indicate standard errors. MEP, motor evoked potential; DS, tactile stimulation to the index finger; C-T interval, conditioning-testing interval.

The effect of the DS on the test MEP was analyzed in 10 participants with attenuation of the TPDS induced by S1-TMS (Table 1). The time course of the modulation of the test MEP amplitude induced by the DS in these 10 participants is shown in Figure 6. There was no apparent modulation of the test MEP induced by the DS. There was no significant difference in the test MEP among the control condition and the five C-T intervals under the DS condition (*F*_(5,45)_ = 0.35, *p* = 0.88).

#### Effect of DS on IHI

**Figure 7:**
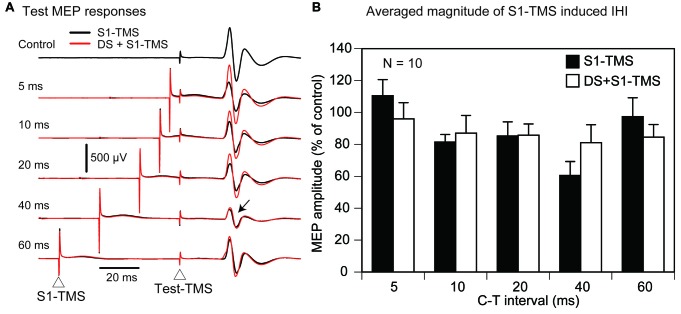
**The averaged test MEP responses (A) and the S1-TMS-induced IHI with and without the DS (B) in the participants with the TP ratio less than 1.0 in Experiment 1.** An arrow points the prominent suppression of the MEP response in both the S1 and DS + S1 conditions **(A)**. Bars indicate means, and error bars indicate standard errors of means **(B)**. MEP, motor evoked potential; S1, primary sensory area; TMS, transcranial magnetic stimulation; DS, tactile stimulation to the index finger; C-T interval, conditioning-testing interval.

The effect of the DS on the S1-TMS-induced IHI was analyzed in 10 participants with attenuation of the TPDS induced by S1-TMS (Table 1). The time course of the IHI under the S1-TMS condition and that under the DS + S1-TMS condition is shown in Figure 7. The IHI was largest with the C-T interval of 40 ms under both conditions, but no apparent difference in the IHI was found between the conditions. Two-way ANOVA revealed a significant difference in the change in the test MEP amplitude among the five C-T intervals (*F*_(4,36)_ = 5.89, *p* < 0.01), but the amplitude was not significantly different between the S1-TMS and DS + S1-TMS conditions (*F*_(1, 9)_ = 0.00, *p* = 0.97) and showed no significant interaction between the two main effects (*F*_(4,36)_ = 1.75, *p* = 0.16).

## Experiment 2

In Experiment 1, the test MEP was inhibited by sub-motor-threshold S1-TMS given 40 ms before the test TMS in the participants with attenuation of the TPDS by the S1-TMS. Nevertheless, the effect of the DS on the S1-TMS-induced IHI was not apparent. In Experiment 2, further investigation was conducted to confirm that the IHI induced by the S1-TMS observed in Experiment 1 is modulated by the tactile input to the S1. In Experiment 1, the interval between the DS and the S1-TMS (conditioning-conditioning [C-C] interval), in which the attenuation of the TPDS was prominent in our preliminary trial, was 30 ms. The N20 of the SEP has been thought to be the activity of the S1, indicating that the afferent volley arrives at the S1 in the period 20 ms after the DS (Allison and Hume, 1981; Emerson and Pedley, 1990; Nuwer et al., 1994). Indeed, in a previous study, the attenuation of the TPDS induced by the S1-TMS was prominent when TMS was given 20 ms after the S1-TMS (Cohen et al., 1991). Thus, in Experiment 2, we formulated an alternative hypothesis—namely, that the S1-TMS-induced IHI is affected by the DS given specifically 20 ms before the S1-TMS, if the S1-TMS-induced IHI is dependent on the status of the S1 modulated by the tactile input.

In order to examine this hypothesis, the S1-TMS-induced IHI was conditioned by the DS with various C-C intervals. In addition, the effect of the DS on the M1-TMS-induced IHI was tested to confirm a hypothesis that the IHI between the M1s is not dependent on the status of the S1 modulated by the tactile input. If this hypothesis is true, the tactile input to the S1 particularly modulates the S1-TMS-induced IHI.

### Materials and Methods

#### Participants

Thirteen healthy humans aged 30.3 ± 1.5 years (11 males and 2 females) participated (Table 1). The participants had no history of neurological disease. All participants were right-handed according to the Edinburgh Handedness Inventory. The methodologies of the EMG recording, DS, test TMS, and the test of the attenuation of the TPDS induced by the S1-TMS were the same as those in Experiment 1.

#### Effect of DS on S1-TMS-Induced IHI

The effect of the DS on the S1-TMS-induced IHI was investigated. The intensities of the DS and S1-TMS were the same as those in the investigation of the IHI in Experiment 1. The S1-TMS was given 40 ms before the test TMS under the S1-TMS condition, because a statistically significant IHI induced by the S1-TMS was present only in this C-T interval in Experiment 1 (Figure 4C). The DS was given at 10, 15, 20, 25, 30, or 35 ms before the S1-TMS (C-C interval) under the DS + S1-TMS condition (Figure 1D). This C-C interval was randomly altered on a trial-by-trial basis. Ten trials were conducted with each C-C interval under the DS + S1-TMS condition. Ten trials solely with the S1-TMS (S1-TMS condition) were randomly inserted between the trials under the DS + S1-TMS condition. The control trial in which only the test TMS was given (control condition) was also randomly inserted between the trials under the S1-TMS and DS + S1-TMS conditions. The number of control trials was 10. The participants closed their eyes with their hands relaxed throughout the experiment. A trigger of the TMS was given when background EMG activity was absent. The inter-trial interval was 10–20 s. The total number of planned experimental trials was 80: 60 trials under the DS + S1-TMS condition, 10 trials under the S1-TMS condition, and 10 trials under the control condition. The trials with a background EMG burst and the trials with an MEP response in the left FDI muscle larger than 50 μV of the amplitude elicited by the S1-TMS were discarded online, and complementary trials were added after the completion of the planned trials.

#### Effect of DS on M1-TMS-Induced IHI

The effect of the DS on the M1-TMS-induced IHI was investigated. The experimental protocol for this investigation was the same as that used to investigate the effect of the DS on the S1-TMS-induced IHI in Experiment 1, except for the intensity and site of the conditioning TMS. The conditioning TMS was given over the hotspot of the left FDI representation. The coil was in the LM position (Harris-Love et al., 2007; Vercauteren et al., 2008; Ni et al., 2009, 2011), where medially directed current was induced in the brain as shown in Figure 1A (Sakai et al., 1997). The intensity of the M1-TMS over the right M1 was 1.2 times the intensity at the resting motor threshold. The M1-TMS was given 40 ms before the test TMS (Figure 1D). The trials with the background EMG burst were discarded online, and complementary trials were added after the completion of the planned trials.

#### Data Analysis

The TP ratio was estimated in all of the participants. The participants in which the test MEP amplitude was within the range of 0.5–1.5 mV were included in the data analysis of the IHI. In the subgroup analysis, only the participants in which the TP ratio was less than 1.0 were included in the data analysis of the IHI. These processes were done, because the significant IHI induced by the S1-TMS was present only when the analysis was conducted for this type of participant in Experiment 1, and we intended to conduct Experiment 2 in the participants whose characteristics were similar to the participants analyzed for the time course of the S1-TMS-induced IHI in Experiment 1. The test MEP amplitude was expressed as a percentage of the average test MEP amplitude under the control condition in each participant.

A *t*-test was conducted to test the difference between the two means. One-way ANOVA was conducted to test the difference in the MEP amplitude among the S1-TMS or M1-TMS condition and the six C-C intervals of the DS + S1-TMS or DS + M1-TMS condition. When one-way ANOVA revealed a significant difference between the means, ANOVA was followed by a multiple comparison (Bonferroni test).

### Results

#### TMS and TP Ratio

The test TMS site was 6.1 ± 0.3 cm left to and 1.0 ± 0.3 cm anterior to the vertex in 13 participants. The test TMS intensity was 76.5 ± 4.4% of the maximum stimulator output. The site of the M1-TMS was 6.2 ± 0.2 cm right to and 0.9 ± 0.3 cm anterior to the vertex. The intensity of the M1-TMS at the resting motor threshold was 65.3 ± 3.1% of the maximum stimulator output. The intensity of the M1-TMS was 78.4 ± 3.7% of the maximum stimulator output. The site of the S1-TMS was 6.2 ± 0.2 cm right to and 2.1 ± 0.3 cm posterior to the vertex. The intensity of the S1-TMS was 94.7 ± 3.3% of the maximum stimulator output. The average TP ratio was 0.81 ± 0.19. The number of participants in whom the TP ratio was equal to 1.0 or more was 3.

#### Effect of DS on S1-TMS-Induced LIHI

**Figure 8:**
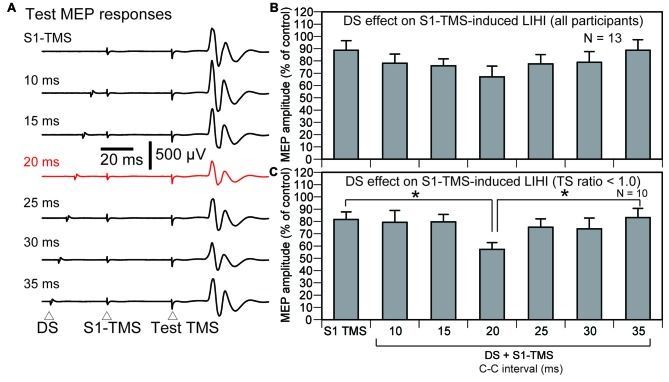
**The averaged test MEP responses (A) and the S1-TMS-induced LIHI with and without the DS across the participants (B) and in the participants with the TP ratio less than 1.0 (C) in Experiment 2.** A red trace indicates the MEP response prominently inhibited **(A)**. The bar at the left side indicates the mean test MEP amplitude under the S1-TMS condition, and the other bars indicate the mean test MEP amplitudes in the various C-C intervals under the DS + S1-TMS condition **(B,C)**. Error bars indicate standard errors. An asterisk indicates significant difference (*p* < 0.05). MEP, motor evoked potential; S1, primary sensory area; TMS, transcranial magnetic stimulation; DS, tactile stimulation to the index finger; C-C interval, conditioning-conditioning interval.

The effect of the DS on the S1-TMS-induced LIHI across all participants is shown in Figure 8B. The average test MEP amplitude under the control condition in these 13 participants was 1.08 ± 0.08 mV. The total number of the trial was 89 ± 2 in this session. The S1-TMS did not decrease the test MEP amplitude significantly compared with the control condition (*p* = 0.08). There was no significant effect of the DS on the S1-TMS-induced LIHI (*F*_(6,72)_ = 2.13, *p* = 0.06). In the subgroup analysis, three participants were excluded from the analysis of the effect of the DS on the S1-TMS-induced LIHI in accordance with the exclusion criteria. Thus, 10 participants (10 males) were included in this analysis (Table 1). The average test MEP amplitude under the control condition in these 10 participants was 1.12 ± 0.08 mV. The S1-TMS significantly decreased the test MEP amplitude compared with the control condition (*p* = 0.02). The MEP amplitude under the S1-TMS condition was 18 ± 6% lower than that under the control condition. The time course of the effect of the DS on the S1-TMS-induced LIHI is shown in Figure 8C. The S1-TMS-induced IHI was enhanced by the DS with the C-C interval of 20 ms (Figure 8A). One-way ANOVA revealed that the test MEP amplitude was significantly different among the S1-TMS condition and the six C-C intervals under the DS + S1-TMS condition (*F*_(6,54)_ = 3.01, *p* = 0.01). A *post hoc* test revealed that the test MEP amplitude under the DS + S1-TMS condition with 20 ms of the C-C interval was significantly smaller than that under the S1-TMS condition and than that under the DS + S1-TMS condition with 35 ms of the C-C interval (*p* < 0.05). The MEP amplitude under the DS + S1-TMS condition with 20 ms of the C-C interval was 30 ± 5% lower than the test MEP amplitude under the S1-TMS condition.

#### Effect of DS on M1-TMS-Induced IHI

**Figure 9:**
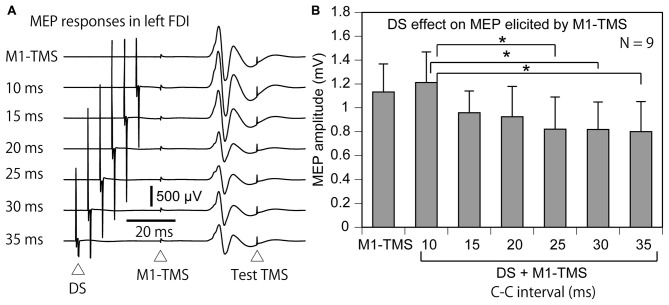
**The average MEP responses in the left FDI muscle preceded by the M1-TMS with and without the DS (A) and the averaged effect of the DS on the left MEP (B) in the participants with the TP ratio less than 1.0.** The bar at the left side indicates the mean MEP amplitude under the M1-TMS condition, and the other bars indicate the mean MEP amplitudes in the various C-C intervals under the DS + M1-TMS condition **(B)**. Error bars indicate standard errors. Asterisks indicate significant differences (*p* < 0.05). MEP, motor evoked potential; M1, primary motor area; TMS, transcranial magnetic stimulation; DS, tactile stimulation to the index finger; C-C interval, conditioning-conditioning interval.

**Figure 10:**
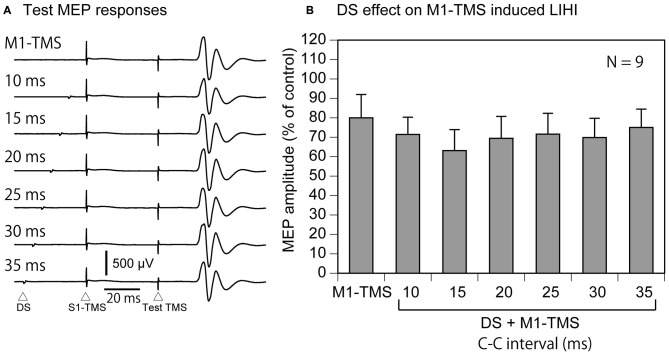
**The average test MEP response preceded by the M1-TMS with and without the DS (A) and the averaged effect of the DS on the M1-TMS-induced LIHI in the participants with the TP ratio less than 1.0 (B).** The bar at the left side indicates the mean MEP amplitude under the M1-TMS condition, and the other bars indicate the mean MEP amplitudes in the various C-C intervals under the DS + M1-TMS condition **(B)**. Error bars indicate standard errors. MEP: motor evoked potential; M1: primary motor area; TMS: transcranial magnetic stimulation; DS: tactile stimulation to the index finger; C-C interval: conditioning-conditioning interval.

Four participants were excluded from the data analysis of the effect of the DS on the M1-TMS-induced LIHI in accordance with the exclusion criteria. Thus, 9 participants (9 males) were included in this analysis (Table 1). The average test MEP amplitude under the control condition was 1.04 ± 0.12 mV in these 9 participants. The total number of the trials were 83 ± 1 in this session. The MEP responses and amplitudes of the MEPs in the left FDI muscle elicited by the M1-TMS during the test of the time course of the LIHI are shown in Figure 9. There was a significant difference in the MEP in the left FDI muscle among the M1-TMS condition and the six C-C intervals under the DS + M1-TMS condition (*F*_(6,48)_ = 3.90, *p* < 0.01). A *post hoc* test revealed that the MEP amplitudes under the DS + M1-TMS condition with 25, 30, and 35 ms of the C-C intervals were significantly smaller than the MEP amplitude under the DS + M1-TMS condition with 10 ms of the C-C interval (*p* < 0.05). Although the difference was not statistically significant, the MEP amplitude under the DS + M1-TMS condition tended to be smaller than that under the M1-TMS condition. This trend must have reflected the SAI. The time course of the effect of the DS on the M1-TMS-induced LIHI is shown in Figure 10. The test MEP amplitude under the M1-TMS condition was 20 ± 11% lower than that under the control condition. The effect of the DS on the M1-TMS-induced LIHI was not apparent. The test MEP amplitude was not significantly different among the M1-TMS condition and the six C-C intervals in the DS + M1-TMS condition (*F*_(6,48)_ = 1.00, *p* = 0.44).

## Discussion

### S1-TMS-Induced Short-Interval IHI

In the present study, the IHI was not induced by sub-motor-threshold S1-TMS given 5–20 ms before the test TMS. The IHI induced by the conditioning TMS around 10 ms before the test TMS is considered to be the short-interval IHI (Chen et al., 2003; Kukaswadia et al., 2005; Ni et al., 2009). Thus, the present finding means that the sub-motor-threshold S1-TMS does not induce the short-interval IHI. This finding was consistent with a previous finding that the short-interval IHI was not induced by the S1-TMS with the intensity below the 2.0 times of the active motor threshold (Ni et al., 2009).

### S1-TMS-Induced LIHI

The S1-TMS given 40 ms after the test TMS did not induce the IHI when estimating the IHI across the participants, but induced that when estimating that in the participants with the TP ratio less than 1.0. The decrease in the test MEP induced by the conditioning TMS over the scalp contralateral to the test TMS site given from 40 to 50 ms before the test TMS is considered to be mediated by the LIHI (Chen et al., 2003; Kukaswadia et al., 2005; Ni et al., 2009). Thus, the decrease in the test MEP induced by the S1-TMS given 40 ms before the test TMS observed in the present study likely reflects the S1-TMS-induced LIHI.

The present finding was consistent with a previous finding that the LIHI was induced by the S1-TMS (Ni et al., 2009). In spite of that, there was an important difference in the experimental procedure between the previous study and our present study. The previous study by Ni et al. (2009) did not confirm whether the MEP response was not elicited by the S1-TMS in each trial. In contrast, in our present study, the MEP response in the left FDI muscle elicited by the S1-TMS was identified in each trial and discarded online so that all the trials analyzed for estimating the S1-TMS-induced IHI did not accompany the MEP response in the left FDI muscle elicited by the S1-TMS. Thus, the present finding is the first to confirm that the S1-TMS induces the LIHI without activation of the conditioning side of the M1 contributing to the contralateral MEP.

Nevertheless, the interpretation of the present finding must be cautiously treated. That is, even the trials with the MEP response elicited by the S1-TMS were discarded from data analysis, this procedure does not guarantee that the whole population of the interneurons in the M1 is not activated by the S1-TMS, because some interneurons in the M1 do not contribute to the MEP, as stated in a previous study that the inhibitory fibers, that had a lower threshold than the S1-TMS, may be activated without producing the contralateral MEP response (Ni et al., 2009). Thus, it is inconclusive that the S1-TMS-induced LIHI is produced without activating whole population of the interneurons in the M1.

### Neural Mechanism Underlying S1-TMS-Induced IHI

The long-lasting IHI of the S1 induced by DS over the hind limb in rats is mediated by the GABA_B_-ergic neurons (Palmer et al., 2012). Moreover, the LIHI induced by M1-TMS in humans is also mediated by the GABA_B_-ergic neurons (Irlbacher et al., 2007). Collectively, the LIHI induced by S1-TMS may also be mediated by the GABA_B_-ergic neurons.

### Sensitivity of TPDS to S1-TMS

Our present finding on the LIHI induced by the sub-motor-threshold S1-TMS was contrary to the previous finding by Ni et al. (2009) and that by Mochizuki et al. (2004). The previous study by Ni et al. (2009) found the S1-TMS-induced LIHI, but the LIHI was absent when the S1-TMS was given at the intensity below the motor threshold according to the experiment on the LIHI induced by the various intensity of the S1-TMS (Ni et al., 2009). Another previous study failed to find the LIHI when the sub-motor-threshold S1-TMS was given 50 ms before the test TMS (Mochizuki et al., 2004). One may speculate that the conflicting findings between our present study and these previous studies may be due to different conditioning coil positions. In our present study, the conditioning coil was in the AP position, but in the previous studies, the coil was in the LM position. The I-waves elicited by the TMS are dependent on the direction of the coil (Sakai et al., 1997; Di Lazzaro et al., 2001). However, the IHI was not affected by the current direction of the conditioning TMS either over the M1 or S1 in previous studies (Chen et al., 2003; Ni et al., 2009). Thus, the conflicting findings between our present study and the previous studies is unlikely due to the position of the conditioning coil.

An important experimental procedure that is different from the previous studies is exclusion of the particular characteristics of the participants. In the present study, the participants without the attenuation of the TPDS induced by the S1-TMS were excluded in the sub-group analysis. This procedure allowed us to obtain apparent LIHI induced by the S1-TMS in the present study. Thus, most likely interpretation for the apparent LIHI induced by the sub-motor-threshold S1-TMS particularly for the sub-group analysis in the present study is that the S1-TMS-induced LIHI occurs only when the S1-TMS affects the pathways mediating the TPDS at the S1.

Moreover, the attenuation of the TPDS by the S1-TMS well correlated with the S1-TMS-induced LIHI. These findings must reflect a fact that both the effects of the DS on the S1-TMS-induced LIHI and the S1-TMS-induced LIHI are dependent on the effect of the S1-TMS on the pathways mediating the TPDS at the S1.

The attenuation of the TPDS induced by the S1-TMS indicates that the S1-TMS interferes the activity of the pathways mediating the TPDS at the S1. Thus, one possible interpretation of the findings on the subgroup analysis is that the pathways mediating the TPDS at the S1 have the inhibitory input to the pathways mediating the S1-TMS-induced LIHI and the pathways mediating the tactile input. Given this is true, in the participants with the attenuation of the TPDS induced by the S1-TMS, inhibitory inputs to the pathways mediating the S1-TMS-induced LIHI and to those mediating the tactile input are decreased, and these decreases cause apparent S1-TMS-induced LIHI and the apparent effect of the DS on the S1-TMS-induced LIHI. Further investigations are needed to elucidate this hypothetical mechanism.

### Effect of DS on LIHI

The M1-TMS-induced LIHI was not enhanced, while the S1-TMS-induced IHI was enhanced by the DS, indicating that the pathways mediating the LIHI induced by the S1-TMS and those mediating the LIHI induced by the M1-TMS have different sensitivity to the DS. A previous study reported that the regression line of the S1-TMS-induced LIHI as a function of the amplitude of the MEP elicited by the S1-TMS was different from that of the M1-TMS-induced LIHI as a function of the amplitude of the MEP response elicited by the M1-TMS, indicating different neural mechanism underlying the S1- and M1-TMS-induced IHIs (Ni et al., 2009). Taken together, the neural pathways mediating the S1-TMS-induced LIHI must not be the same as the pathways mediating the M1-TMS-induced LIHI.

In a previous study, the activity of the M1 was decreased by the DS over the ipsilateral fingers (Hlushchuk and Hari, 2006). Thus, one may speculate that the enhancement of the S1-TMS-induced LIHI caused by the DS reflects the direct inhibition of the test MEP induced by the DS. Nevertheless, this speculation is unlikely. In Experiment 1, the test MEP was not modulated when only the DS was given to the test MEP side, as consistent with a previous finding that the electrical stimulation of the ulnar nerve did not modulate the ipsilateral test MEP (Ni et al., 2009). The S1-TMS was given from 5 to 60 ms before the test TMS, and the DS was consistently given 30 ms before the S1-TMS. That is, the test MEP was not directly modulated by the DS given from 35 to 90 ms before the test TMS. In Experiment 2, the S1-TMS-induced LIHI was enhanced by the DS given 20 ms before the S1-TMS. In this experiment, the S1-TMS was given 40 ms before the test TMS. That is, the DS enhanced the S1-TMS-induced LIHI when the DS was given 60 ms before the test TMS. Thus, the interval between the DS and the test TMS that caused the enhancement of the IHI induced by the DS in Experiment 2 was within the range of the interval between the DS and the test TMS in which the DS alone did not modulate the test MEP in Experiment 1. Accordingly, the enhancement of the S1-TMS-induced LIHI by the DS is not due to the direct inhibition of the test MEP solely by the DS given over the finger ipsilateral to the test MEP side.

The N20 of the SEP reflects the activity of the S1 (Allison and Hume, 1981; Nuwer et al., 1994). That is, the latency of the change in the activity of the S1 when the DS is given over the MN at the wrist must be around 20 ms. In the present study, the C-C interval of the DS causing the enhancement of the S1-TMS-induced LIHI was 20 ms. Thus, the time taken for the conduction of the afferent volleys of the MN from the wrist to the S1 corresponds well to the appropriate C-C interval of the DS that enhanced the S1-TMS-induced LIHI. In the other words, the S1-TMS-induced LIHI is enhanced only when the S1-TMS is given when the tactile afferents produced by the digit stimulation just arrive at the S1. This suggests that the S1-TMS-induced LIHI is dependent on the activity of the S1 that is enhanced by the tactile input.

### Hypothetical Interaction Between Pathways and Events

Several present findings imply the interaction between the pathways and events related to the IHI. The LIHI was induced by the TMS over the S1, indicating the S1-TMS activates the pathways mediating the LIHI. The DS given 20 ms before the S1-TMS increased the IHI, indicating that the tactile input to the S1 enhances the pathways mediating the S1-TMS-induced IHI. In contrast, the S1-TMS attenuates the TPDS, indicating that the pathways mediating the TPDS are inhibited by the TMS given over the S1. The LIHI was positively correlated with the attenuation of the TPDS induced by the S1-TMS, and was apparent only in the participants with the attenuation of the TPDS induced by the S1-TMS. Accordingly, it is likely that the pathways mediating the TPDS have the inhibitory inputs to the pathways mediating the S1-TMS-induced IHI. Tactile input to the S1 enhanced the S1-TMS-induced IHI, and the enhancement was apparent in the participants with attenuation of the TPDS by the S1-TMS. Thus, the pathways mediating the TPDS may have the inhibitory inputs to the pathways mediating the tactile input to the S1. Further investigations are needed for elucidating these hypothetical mechanisms.

### Laterality and Handedness

In the present study, the LIHI of the left M1 was induced by the TMS over the contralateral S1. The testing and conditioning TMS sides were same as the previous study by Ni et al. (2009). However, the S1-TMS-induced LIHI observed in the present study may be different between the hemispheres, because the M1-TMS-induced IHI is different between the hemispheres in the right handers (Netz et al., 1995). Moreover, in the present study, the S1-TMS-induced IHI was investigated on the right handers. However, the S1-TMS-induced LIHI observed in the present study may be dependent on handedness, because of a previous finding that the laterality of the M1-TMS-induced IHI is dependent on the handedness (Bäumer et al., 2007). These issues must be investigated in future studies.

### Methodological Consideration

The S1-TMS-induced LIHI was not found when the data analysis was conducted across all participants. In some previous studies, the locus of the S1 was identified using anatomical MRI (Meehan et al., 2008; Ni et al., 2009). On the other hand, in the present study, the S1-TMS was given over the site 3 cm posterior to the hotspot of the FDI representation, because this site has been shown to be the appropriate site of the TMS over the S1 (Harris et al., 2002; McKay et al., 2003; Koch et al., 2006; Palomar et al., 2011). The anatomical accuracy of the TMS site of the S1 in the present study must have been less than that in the previous studies using anatomical MRI. This may have been the cause of the variable results of the S1-TMS-induced LIHI among the participants. Nevertheless, in the present study, the functional efficiency of the S1-TMS on the TPDS was estimated. The S1-TMS-induced LIHI was apparent when the participants for estimating the IHI were filtered by the attenuation of the TPDS induced by the S1-TMS. Thus, the attenuation of the TPDS induced by the S1-TMS may be a useful measure that confirms the functional efficiency of the TMS over the S1 and the accuracy of the determination of the S1 locus without anatomical MRI.

### Summary

The LIHI was not induced by the S1-TMS without activation of the conditioning side of the M1 contributing to the contralateral MEP when the IHI was estimated across the participants, but was induced by that when the LIHI was estimated in the participants with the attenuation of the TPDS induced by the S1-TMS. The S1-TMS-induced LIHI was positively correlated with the attenuation of the TPDS induced by S1-TMS, indicating that the S1-TMS-induced LIHI is dependent on the effectiveness of the S1-TMS on the pathways mediating the TPDS at the S1. The S1-TMS-induced LIHI was enhanced when S1-TMS was given in the period in which the tactile afferent volley produced by the digit stimulation just arrived at the S1, while the LIHI induced by above-motor-threshold TMS over the contralateral M1 was not enhanced by the tactile input. These findings indicate that the S1-TMS-induced LIHI is dependent on the status of the S1 modulated by the tactile input, and the pathways mediating the sub-motor-threshold S1-TMS-induced LIHI are not the same as the pathways mediating the above-motor-threshold M1-TMS-induced LIHI.

## Author Contributions

YI and KH, study design, conducted the experiment, analyzed the data, writing article. YJ, HM and AK, conducted the experiment.

## Conflict of Interest Statement

The authors declare that the research was conducted in the absence of any commercial or financial relationships that could be construed as a potential conflict of interest.
